# A Survey on HIV/AIDS-Related Knowledge, Attitudes, Risk Behaviors, and Characteristics of Men Who Have Sex with Men among University Students in Guangxi, China

**DOI:** 10.1155/2020/7857231

**Published:** 2020-06-14

**Authors:** Jingzhen Lai, Peijiang Pan, Yulun Lin, Li Ye, Long Xie, Yuan Xie, Bingyu Liang, Fangling Zheng, Rongfeng Chen, Liufang Wen, Yuxin Luo, Hao Liang, Junjun Jiang

**Affiliations:** ^1^Guangxi Key Laboratory of AIDS Prevention and Treatment & Guangxi Universities Key Laboratory of Prevention and Control of Highly Prevalent Disease, School of Public Health, Guangxi Medical University, Nanning, Guangxi 530021, China; ^2^Guangxi Collaborative Innovation Center for Biomedicine, Life Sciences Institute, Guangxi Medical University, Nanning, Guangxi 530021, China; ^3^School of Public Health, Guangxi Medical University, Nanning, Guangxi 530021, China; ^4^Institute of Cellular Medicine, Medical School, Newcastle University, NE2 4HH, UK

## Abstract

In recent years, the prevalence of human immunodeficiency virus (HIV) infection among Chinese university students has increased significantly, and HIV transmission among men who have sex with men (MSM) comprises more than half of the new cases. There is still a lack of research investigating the incidence of male-to-male sex, the attitudes towards MSM, and the awareness of HIV/AIDS among university students in Guangxi, one of the HIV high-risk areas in China. Therefore, we performed a cross-sectional investigation among 578 male students, recruited by stratified sampling, in universities in Nanning, Guangxi, between January 2016 and March 2017. Researcher-administered anonymous questionnaires were completed. Self-recognition as MSM was found in 8.48% of the subjects. Compared with non-MSM, university student MSM included more people over the age of 20 (OR = 4.95), had less migration from other districts of Guangxi (OR = 0.26), and the majority were nonmedical students (OR = 8.99). In total, 63.25% of the male student participants reported a lack of acceptance of MSM, while 35.47% acknowledged barriers between themselves and acquaintances who were MSM. Overall, 67.30% of the subjects correctly answered questions related to AIDS knowledge. The proportion of MSM subjects who answered the AIDS-related questions completely correctly was significantly lower than that of non-MSM subjects (42.86% vs. 69. 57%, respectively, OR: 0.33), but the self-recognition risk of MSM was significantly higher than that of non-MSM (OR = 2.59). Risky behaviors associated with HIV infections, including smoking, alcohol consumption, drug abuse, and inconsistent condom use, were significantly higher among the MSM participants. The percentages of student's willingness to accept MC and PrEP were 70.93% and 77.51%, respectively. These results raise the alarm that university student MSM in Guangxi, China, require urgent public attention and more effective health education, including the education on MC and PrEP.

## 1. Introduction

Acquired Immune Deficiency Syndrome (AIDS) has been an epidemic worldwide since the 1980s. The Joint United Nations Programme on HIV and AIDS (UNAIDS) proposed a global goal: end the AIDS epidemic as a public health threat by 2030. However, 37.9 million people were living with human immunodeficiency virus (HIV) by the end of 2018 [1]. The reported number of HIV infections in China was 861042 by the end of 2018 [2]. There is a long way to go before HIV/AIDS is under control by 2030.

Among all high-risk populations of HIV infection, men who have sex with men (MSM) have been found to have an increased risk of HIV incidence [3]. High risky behaviors including unprotected anal intercourse (UAI) and multiple sexual partners made MSM at high risk of HIV infection. As sexual transmission has become the main mode of HIV transmission, the proportion of MSM in new HIV cases has been increasing each year. The proportion of estimated new HIV cases among MSM increased from 2.5% in 2006 to 21.4% in 2013, according to the 2014 China AIDS Response Progress Report [4]. Among the reported cases of HIV infection in China in 2015, 28.4% were transmitted through male-to-male intercourse [5]. In some developed countries, the percentage of HIV infection transmitted through male-to-male sex is much higher than that in China. For instance, MSM accounted for 71.5% of the total estimated newly diagnosed HIV positives in Japan, 67% in the US, and approximately 54% in Canada in 2014 [6–8].

A study focused on male-to-male sex as a risky sexual behavior in relation with HIV prevalence in urban China found a significant proportion of MSM among college students [9]. Furthermore, the number of HIV newly diagnosed college students presented a 30% to 50% annual growth rate over the past several years [10]. A study also reported a significantly increased HIV epidemic trend that the HIV prevalence among high school and college student MSM in China was 3.0% in 2003-2006, 4.5% in 2007-2008, and 6.8% in 2009-2010 [11]. University students are sexually active and have strong desires regarding sexual exploration, resulting in a large amount of unprotected sexual behaviors, including homosexual behavior. As reported by the Guangxi Centers for Disease Control and Prevention (CDC), among the newly reported HIV cases in 2015, 126 were identified as students. Among the 126 student HIV positives, 89.68% were male, and 59.52% were acquired through homosexual behavior [12]. The percentage of male-to-male HIV acquisition among student HIV positives is much higher than that among the national reported cases in China [2, 4, 5]. This data is even higher than the quoted percentage of HIV infections among Canadian MSM [8]. Therefore, the HIV epidemic among university students in China has been recognized as a serious problem. Nevertheless, the proportion of MSM among university students in China remains unknown.

Guangxi, a southwestern province of China, is one of the highest-prevalence provinces of HIV infection in China [13]. Although the steadily increasing HIV prevalence of MSM in Guangxi has been observed [14], the percentage of MSM in Guangxi has rarely been reported, and the number of MSM among university students in Guangxi was even less reported.

While condom use is considered one of the most effective measures to prevent HIV infection, among those who are less consistent in condom usage, such as MSM, more interventions are needed to enhance HIV prevention, for example, male circumcision (MC) and pre-exposure prophylaxis (PrEP). Three randomized controlled trials have demonstrated that MC can reduce the risk of heterosexual HIV acquisition by up to 60% [15–17], and MC may reduce the risk of homosexual HIV acquisition among MSM who primarily practice insertive anal sex [18]. PrEP is the use of one or a combination of antiretroviral drugs in HIV-negative individuals to reduce their risk of infection. It has been indicated that daily PrEP is a promising strategy to reduce HIV infection [19–21]. In 2014, the US CDC recommended PrEP as an option for persons at risk of acquiring HIV [22]. To date, PrEP has not been popularized in China, and the studies on PrEP in China have been mostly focused on the willingness to use PrEP [23–25]. Although a previous report has indicated university students' acceptance of MC [26], there is a lack of studies suggesting to what extent PrEP is accepted by male university students (MUSs).

The purpose of this study is to provide scientific guidance for HIV/AIDS prevention publicity and education in the MUS population, especially the high-risk MSM population in Guangxi, China, and to promote MC and PrEP among the MUSs. We recruited MUSs as the subjects to investigate the proportion and characteristics of MSM among university students. Meanwhile, the acceptance of MC and PrEP and its associated factors were investigated.

## 2. Materials and Methods

### 2.1. Study Design and Population

A cross-sectional study was conducted among MUSs in five universities in Nanning, the capital of Guangxi, China, between January 2016 and March 2017. Considering the feasibility of investigation, about 80% of the participants were sampled from Guangxi Medical University, and the other 20% of the participants were sampled from Guangxi University, Guangxi University for Nationalities, Guangxi University of Chinese Medicine, and Guangxi Arts University. To be eligible for the study, participants should be (1) male, (2) students from the five universities, (3) aged 16 and above, and (4) able to give verbal (in Mandarin) and written consent. The sampling method used for the universities was convenient sampling. Students from selected universities were sampled by random sampling. The student ID number of all male students from the selected universities was listed in a table. After that, the students were randomly sampled from the different universities using a random number table. The sample size was calculated based on the equation, as follows:

(1) $$\begin{array}{c} \;N=\frac{Z_{\alpha }^{2}\times pq}{d^{2}}, \end{array}$$

where *Z*_*α*_ means the standard normal distribution bound, *α* was set as 0.05, and *Z*_*α*_ was set as 1.96. *p* denotes the percentage of MSM among MUSs, and *q* = 1 − *p*. *d* is the allowable error, which was set as 0.15*p*. We did not know the percentage (*p*) of MSM among MUSs. Therefore, we estimated the percentage of MSM among MUSs to be 28.4% based on the fact that 28.4% of reported cases of HIV infection in China in 2015 were transmitted via male-to-male intercourse [5]. The sample size was chosen as 431 based on the equation. We took the possibility of no response to the questionnaire into consideration and set the sample size at 500 participants. All subjects were MUSs, including undergraduates and postgraduates. All participants were informed of the study and signed informed consent before participation. The study was approved by the Human Research Ethics Committee of Guangxi Medical University.

### 2.2. Data Collection

The survey was conducted via an interviewer-administered anonymous questionnaire see (available here) that was composed of several parts, including the student's sociodemographic background, knowledge regarding HIV/AIDS, attitude towards MSM and HIV/AIDS, risk behavior related to HIV transmission, and acceptance of MC and PrEP for prevention of HIV infection. Students with a medical education background, including Clinical Medicine, Stomatology, Pharmacology, Preventive Medicine, and Social Health Management, were defined as being involved in a medical specialty, since such a background produces systematic HIV/AIDS education exclusively.

Knowledge and attitudes towards MSM were investigated by asking 4 questions. Comprehension of MSM means the overall understanding of male homosexuality. The reply options included “unchangeable,” “treatable,” “infectious,” and “normal.” “Unchangeable” means MSM are born with homosexuality and cannot be changed. “Treatable” means that homosexuality is kind of a disease and can be treated by medicine. “Infectious” means that male homosexual behavior can spread in the form of infection. “Normal” means MSM are a normal population with normal psychological states. “Attitude towards MSM around” means the attitudes participants had when they discovered that some of their friends were MSM. Respondents who were interested in MSM and wanted to know more about them answered “interested.” “Indifferent” refers to those respondents who felt indifferent and did not care when finding out that some of their friends were MSM. “Uncomfortable” means the respondents would continue their friendship with MSM but feel uncomfortable. “Aloof” means the respondents choose to stay away from MSM. When asked, “what would you do if you fell in love with a man,” respondents were given three options, including staying in love, refraining from touching a man, or panicking. HIV/AIDS knowledge was assessed using an 8-item questionnaire designed by the Chinese CDC [27]. Giving all eight correct answers was defined as 100% correct on HIV/AIDS knowledge. MC/PrEP acceptance was measured by self-reported “yes” or “no” on the questionnaire.

### 2.3. Statistical Analysis

All questionnaires were checked and verified, and there were no logical errors. The data were double entered into EpiData 3.1 (the EpiData Association, Odense, Denmark) by two different researchers and examined as a consistency check. The participants were divided into two groups, MSM and non-MSM. Bivariate analyses were performed to find variables for sociodemographic background, knowledge regarding HIV/AIDS, attitude towards MSM and HIV/AIDS, risk behaviors related to HIV transmission, and acceptance of MC and PrEP between MSM and non-MSM. Bivariate logistic regression models were utilized to find the independent factors associated with MC/PrEP acceptance. All statistical analyses were conducted by IBM SPSS Statistics 23.0 (IBM Corp, Armonk, NY, USA). All tests were two-sided, and *p* < 0.05 was considered statistically significant.

## 3. Results

### 3.1. Demographic Characteristics

A total of 591 MUSs were recruited to take part in the questionnaire survey, of which 13 did not submit. Thus, 578 students agreed to participate and completed the questionnaire (Table 1). The proportion of MSM was 8.48% (49/578). The age of the participants ranged from 17 to 27 years (median: 21, interquartile range: 20–22). Subjects in this study were divided into two groups at the age of 20, those aged 20 and under (46.37%) and those aged over 20 (53.6%). As for the ethnicity, most of the participants (75.43%) were of Han nationality. Of all respondents, 86.33% were registered as residents in Guangxi province, and among them, 21.11% were Nanning residents. Of all respondents, the majority of the students (81.14%) were majoring in Medicine, 40.66% were freshmen, and 34.4% were juniors. The distribution of the participants is shown in Table 1. The participants in this study were divided into two groups based on self-identification as MSM or not. Table 1 shows some sociodemographic characteristics that were found to be associated with MSM. MSM tended to be above 20 years of age (OR = 4.95), non-Medicine majors (OR = 8.99), and in their senior years. In addition, according to registered residence, MSM were less likely to be from non-Nanning cities (OR = 0.26), and MSM had a longer living time in Nanning city.

### 3.2. Knowledge of MSM and Attitude toward MSM

All participants were investigated about their knowledge of and attitude toward MSM. Of all 578 MUSs, 47.75% thought that MSM were normal, but 63.32% showed negative responses to the option of accepting MSM. Nearly 50% of the students felt no difference when there was an MSM around, compared with slightly fewer respondents who felt uncomfortable. In addition, 75.96% of the students chose an unpleasant response to the item evaluating falling love with a male (Table 2).

### 3.3. Differences in Knowledge, Attitude, and Risk Behaviors of HIV/AIDS between MSM and Non-MSM

Concerning HIV/AIDS-relevant knowledge, 67.30% of all students correctly answered all of the items. In comparison with non-MSM participants, the percentage of MSM subjects obtaining the full score of correct HIV/AIDS knowledge was significantly lower (42.86% vs. 69.57%, respectively, OR: 0.33). More than half (53.06%) of MSM said that they were not afraid of HIV/AIDS. The proportion of people who felt that they were at risk of HIV infection varied among different groups. A higher proportion of MSM considered that they were at risk than that of non-MSM (OR = 2.60).  Table 3 also shows the risky behaviors, such as smoking, alcohol consumption, and drug abuse, that make students more likely to become infected with HIV if they engaged in MSM. In addition, of the 134 participants who had sex experience, only 19.4% had used condoms consistently in the last six months. Among MSM students, only 18.37% MSM used condoms consistently.

### 3.4. Acceptance of MC and PrEP

Although only 20.07% of MUSs were circumcised, 70.93% of the students expressed their willingness to undergo the procedure order to prevent HIV infection (shown in Table 4). While 21.28% of the students had prior knowledge of PrEP, only a small proportion (3.46%) had applied this measure. Similar to MC, PrEP was generally accepted by 77.51% of MUSs. The proportion of MSM who had undergone MC surgery was much higher than that of non-MSM (14.29% vs. 9.07%, OR: 2.55). In contrast, the proportion of MSM who were willing to undergo MC was lower than that of non-MSM (38.78% vs. 73.91%, OR: 0.22). Concerning the implementation of PrEP, the proportion of users in MSM was significantly higher than that in non-MSM (20.41% vs. 1.89%, respectively, OR: 13.31), although the acceptability of PrEP was similar between the MSM and non-MSM groups.

### 3.5. Factors Associated with Acceptance of MC and PrEP

Table 5 shows the results of bivariate logistic regression analysis of variables, including demographic characteristics, knowledge and attitude towards HIV/AIDS and MSM, and HIV-related risk behaviors associated with MC and PrEP acceptance among MUSs. Acceptance of MC/PrEP (a score of 1 for accepting MC/PrEP, 0 for the opposite choice) was defined as the dependent variable. MC surgery experience was entered into the model when the factors associated with MC acceptance were analyzed. Prior knowledge of PrEP and application experience of PrEP were entered into the model when the factors related to PrEP acceptance were analyzed. The factors of registered residence other than Guangxi province, medicine background, and previous MC surgery experience were found to facilitate the acceptability of MC as a measure for the prevention of HIV infection. Self-identification as MSM and unknown MC surgery experience were found to be independent impeditive factors to the acceptance of MC. The Zhuang ethnic minority was found as the independent promoting factor of PrEP acceptance. Living in Nanning for more than 2 years, thinking MSM is infectious, alcohol consumption < 250 ml/day, and the experience in PrEP implementation were found to be independent impeditive factors to the acceptance of PrEP (Table 5).

## 4. Discussion

In China, sexually active male university students are becoming an important bridge to spread HIV between MSM and the general population [5, 12]. Given that MSM are more at risk to HIV infection, to investigate the proportion of MSM among MUSs is fundamental to assess the prevalence of HIV/AIDS. Meanwhile, MC and PrEP are considered to be effective HIV prevention strategies and need to be promoted among high-risk populations, including university students who seldom consistently use condoms [20]. Although some previous studies have shown the factors related to the acceptance of MC among MUSs in China, to our knowledge, this is the first study in China to explore factors associated with acceptance of PrEP among MUSs.

This study took male university students as the target population, and its representativeness will provide certain value for further epidemiological studies. Understanding the characteristics of this population will contribute to future efforts to curb the AIDS epidemic in Chinese universities. The 8.5% of students identified as MSM in this study was higher than that previously reported from two universities in Zhejiang province, China (3.7%) [28].

The present study found some interesting phenomena. MSM had less knowledge of HIV/AIDS; were more likely to use cigarettes, alcohol, and drugs; and were less likely to consistently use condoms. Inconsistent condom use, smoking, drinking, and drug abuse have been considered to be strongly associated with HIV risk. Therefore, our findings indicate that low levels of HIV/AIDS knowledge and blind fearlessness may interact and jointly contribute to the spread of HIV/AIDS among MSM students in China. Furthermore, sexual abuse resulting from alcohol and drug abuse has further contributed to the HIV/AIDS epidemic. In addition, MSM are more likely to have sex without condom use, since having sex with a condom reduces sexual pleasure. Hence, a notable rate of condom abandonment is due to the pursuit of sexual satisfaction among MSM. Our study suggests that effective interventions such as MC and PrEP could be implemented for the prevention and control of HIV/AIDS among the student MSM population, which is an emerging high-risk population for HIV transmission currently in China. Through the implementation of these interventions, the risky behaviors mentioned above could be placed under control along with the improvement in essential knowledge regarding HIV/AIDS.

MC and PrEP are complementary measures used to control the spread of HIV. In this study, among MUS participants, the overall acceptance rate of MC was 70.9%, which is much higher than that (55.2%) in the same region and population in 2015 [26]. This MC acceptance rate is also higher than those reported among black Africans in South Africa (45.7%), the Rakai population in Uganda (27%), young black MSM in the US (50%), and male rural-to-urban migrants in western China (37.3%) [29–33]. Our study shows that people who were not registered residents of Guangxi, had a medicine background, and had an MC surgery were more likely to accept MC. The high acceptance of MC among non-Guangxi-registered participants was consistent with previous reports in China [34]. The medical education background helps to improve the acceptance rate to MC, because they understand the benefits of MC in preventing HIV infection. On the other hand, our results indicate a low MC uptake/acceptance amongst MSM, which is contrary to conclusions from other studies [35]. Some reports suggest that in a population with at least a 50% MC acceptance rate, the MC program would be effective in preventing HIV transmission [31, 34], which is higher than the 38.8% of MC acceptance rate observed in our study.

A previous study recruited uncircumcised male students from some medical schools in Western China to explore the acceptability of MC and associated factors, revealing some reasons why the MC acceptance rate is higher among medical students than among other general populations [26]. This report shows that a medical education background was associated with MC acceptance, as well as expected improvement in sex hygiene (67.3%), positive prevention of HIV and other sexually transmitted diseases (STDs) (46.3%), and, interestingly, increased sexual satisfaction (65.3%) [26]. To some extent, this is consistent with our observation that medical students have a more positive attitude towards MC acceptance.

Regarding PrEP, a number of studies have explored the acceptance of PrEP and related factors in different HIV high-risk populations, including the MSM population. Nevertheless, little information is available about the willingness of implementing PrEP for HIV prevention among the university students in China. Our study reported that 77.5% of MUS participants were willing to accept PrEP, which is higher than those previously reported in different places in the world, such as 39% among Australian gay and bisexual men in 2015 [36], 64% among HIV-negative MSM in Western China in 2010, 48% in Washington DC in 2014, 60% in Miami in 2014, 48% in Baltimore in 2011, 55.4% in New York in 2012, and 61% in 20 US cities monitored by the National HIV Behavioral Surveillance system in 2014 [25, 37–40]. In addition, it was found in this study that participants from the Zhuang ethnic minority were more likely to accept PrEP, indicating that ethnicity may be a related factor. Furthermore, our study demonstrates that living in Nanning for more than 2 years, thinking that MSM is infectious, alcohol consumption < 250 ml/day, and the experience of PrEP are barriers to PrEP use. No studies so far have reported the effect of alcohol consumption on PrEP implementation. The withdrawal rate of PrEP, which occurs in HIV-negative individuals using PrEP, can be defined as the adherence problem of PrEP. Studies of PrEP compliance have identified three key factors that are associated with the barrier of the initiation of PrEP and motivation to adhere to the medication [41]. The first one is skepticism about the effectiveness of PrEP in combination with other prophylaxes to prevent HIV infection [41]. The second barrier comes from the stigma associated with PrEP, such as the inherent stigma associated with HIV infection [41, 42]. The third barrier is its unpleasant side effects [41]. We think that there are similar barriers in China; thus, the use of PrEP is not common among MUSs in China yet. Nevertheless, addressing the barriers negatively associated with adherence motivation would help to implement PrEP.

Our study is subject to several limitations. Firstly, for some sensitive questions, the answers of some participants might not match their actual behaviors. Secondly, the participants were locally enrolled from Guangxi province. Small spatial distribution of the samples as well as no significant difference in ethnicity and socioeconomic status limits our findings regarding representing the whole nation of China. Thirdly, our findings are based on self-reported data, which might be affected by recall bias. Finally, the complex sampling method used to recruit MUS may have affected the generalizability of our data, which was not taken into account. Considering the feasibility of investigation, about 80% of the participants were sampled from Guangxi Medical University. A lot of medical students included may have also led to selection bias.

## 5. Conclusion

This study reveals the proportion of university student MSM in Guangxi, China. Concerning HIV risky behaviors, this study found MSM students were more likely to have inconsistent condom use, smoking, alcohol consumption, and drug abuse. These HIV high-risk behaviors among university students suggest the urgent need for increased public attention and more effective health education, including that on MC and PrEP. In particular, educational information or supplementary information about PrEP is urgently needed to address the side effect concerns, which may attenuate MSM willingness to accept PrEP.

## Figures and Tables

**Table 1:** Demographic characteristics of MSM and non-MSM in university and odds ratios.

Variables	Total (%)	MSM (%)	Non-MSM (%)	OR (95% CI)	*p* value
Age					
≤20	268 (46.37)	8 (16.32)	260 (49.15)	1.00	
>20	310 (53.63)	41 (83.67)	269 (50.85)	4.95 (2.28, 10.77)	<0.001
Nationality					
Han	436 (75.43)	42 (85.71)	394 (74.48)	1.00	
Zhuang	114 (19.72)	5 (10.20)	109 (20.60)	0.43 (0.17, 1.11)	0.082
Other minorities	28 (4.84)	2 (4.08)	26 (4.91)	0.72 (0.17, 3.15)	0.664
Registered residence					
Nanning city	122 (21.11)	22 (44.90)	100 (18.90)	1.00	
Other cities in Guangxi	377 (65.22)	20 (40.82)	357 (67.49)	0.26 (0.13, 0.49)	<0.001
Provinces besides Guangxi	79 (13.67)	7 (14.29)	72 (13.61)	0.44 (0.180, 1.09)	0.076
Time living in Nanning					
1 year	263 (45.50)	14 (28.57)	249 (47.07)	1.00	
1~2 years	157 (27.16)	8 (16.33)	149 (28.17)	0.96 (0.39, 2.33)	0.919
>2 years	158 (27.34)	27 (55.10)	131 (24.76)	3.67 (1.86, 7.23)	<0.001
Major					
Medical	469 (81.14)	19 (38.78)	450 (85.07)	1.00	
Nonmedical	109 (18.86)	30 (61.22)	79 (14.93)	8.99 (4.83, 16.76)	<0.001
Grade					
One	235 (40.66)	4 (8.16)	231 (43.67)	1.00	
Two	81 (14.01)	8 (16.33)	73 (13.80)	6.33 (1.85, 21.63)	0.003
Three	199 (34.43)	20 (40.82)	179 (33.84)	6.45 (2.17, 19.21)	0.001
Four	45 (7.79)	12 (24.49)	33 (6.24)	21.00 (6.40, 68.95)	<0.001
Five	4 (0.69)	1 (2.04)	3 (0.57)	19.25 (1.63, 227.49)	0.019
Postgraduate	14 (2.42)	4 (8.16)	10 (1.89)	23.10 (5.03, 106.00)	<0.001
Total	578 (100.00)	49 (8.48)	529 (91.52)		

**Table 2:** Knowledge of and attitude toward MSM among MUSs.

Variables	Number	Proportion (%)
Comprehension of MSM		
Unchangeable	159	27.51
Treatable	74	12.80
Infectious	69	11.94
Normal	276	47.75
Can you accept MSM?		
Yes	212	36.68
No	366	63.32
Attitude towards MSM around		
Interested	44	7.61
Indifferent	256	44.29
Uncomfortable	205	35.47
Aloof	73	12.63
What would you do if you fell in love with a male?		
Stay in love	139	24.05
Refrain from touching a man	188	32.53
Panic	251	43.43
Total	578	100.00

**Table 3:** Knowledge, attitude, and risk behaviors for HIV/AIDS of MSM versus non-MSM in university.

Variables	Total	MSM (%)	Non-MSM (%)	OR (95% CI)	*p* value
100% correct on HIV/AIDS-related knowledge					
No	189 (32.70)	28 (57.14)	161 (30.43)	1.00	
Yes	389 (67.30)	21 (42.86)	368 (69.57)	0.33 (0.18, 0.60)	<0.001
Are you afraid of AIDS?					
No	120 (20.76)	26 (53.06)	94 (17.77)	1.00	
A little	200 (34.60)	7 (14.29)	193 (36.48)	0.13 (0.06, 0.31)	<0.001
Very	258 (44.64)	16 (32.65)	242 (45.75)	0.24 (0.12, 0.47)	<0.001
Risk of HIV infection					
Never	244 (42.21)	25 (51.02)	219 (41.40)	1.00	
Low	299 (51.73)	16 (32.65)	283 (53.50)	0.50 (0.26, 0.95)	0.035
High	35 (6.06)	8 (16.33)	27 (5.10)	2.60 (1.07, 6.33)	0.036
Was there health education on homosexuality at school?					
No	263 (45.50)	23 (46.94)	240 (45.37)	1.00	
Yes	315 (54.50)	26 (53.06)	289 (54.63)	0.94 (0.52, 1.69)	0.833
Frequency of smoking					
Never	426 (73.70)	19 (38.78)	407 (76.94)	1.00	
Occasional	98 (16.96)	15 (30.61)	83 (15.69)	3.87 (1.89, 7.93)	<0.001
<10 cigarettes/day	46 (7.96)	12 (24.49)	34 (6.43)	7.56 (3.39, 16.88)	<0.001
10–20 cigarettes/day	4 (0.69)	2 (4.08)	2 (0.38)	21.42 (2.86, 160.39)	0.003
>20 cigarettes/day	4 (0.69)	1 (2.04)	3 (0.57)	7.14 (0.71, 71.90)	0.095
Frequency of alcohol consumption					
Never	255 (44.12)	17 (34.69)	238 (44.99)	1.00	
Occasional	273 (47.23)	17 (34.69)	256 (48.39)	0.93 (0.46, 1.86)	0.837
<250 ml/day	15 (2.60)	8 (16.33)	7 (1.32)	16.00 (5.18, 49.40)	<0.001
250–500 ml/day	16 (2.77)	3 (6.12)	13 (2.46)	3.23 (0.84, 12.44)	0.088
>500 ml/day	19 (3.29)	4 (8.16)	15 (2.84)	3.73 (1.12, 12.49)	0.033
Varieties of illegal injected drugs					
No	510 (88.24)	25 (51.02)	485 (91.68)	1.00	
Heroin	4 (0.69)	2 (4.08)	2 (0.38)	19.40 (2.62, 143.46)	0.004
Methamphetamine	59 (10.21)	19 (38.78)	40 (7.56)	9.22 (4.68, 18.15)	<0.001
Cocaine	1 (0.17)	1 (2.04)	0 (0.00)	3.13*E*10 (0.00, -)	1.000
Other	4 (0.69)	2 (4.08)	2 (0.38)	19.40 (2.62, 143.46)	0.004
Frequency of condom use in the last six months (*n* = 134)					
Every time	26 (19.40)	9 (18.37)	17 (20.00)	1.00	
Sometimes	44 (32.84)	28 (57.14)	16 (18.82)	3.31 (1.20, 9.12)	0.021
Never	10 (7.46)	7 (14.29)	3 (3.53)	4.41 (0.91, 21.30)	0.065
No sex experience	54 (40.30)	5 (10.20)	49 (57.65)	0.19 (0.06, 0.66)	0.008

**Table 4:** Acceptance of MC and PrEP among MSM and non-MSM in university.

Variables	Total	MSM (%)	Non-MSM (%)	OR (95% CI)	*p* value
MC surgery					
No	462 (79.93)	25 (51.02)	437 (82.61)	1.00	
Yes	55 (9.52)	7 (14.29)	48 (9.07)	2.55 (1.05, 6.21)	0.039
Unknown	61 (10.55)	17 (34.69)	44 (8.32)	6.75 (3.39, 13.46)	<0.001
Acceptance of MC					
No	168 (29.07)	30 (61.22)	138 (26.09)	1.00	
Yes	410 (70.93)	19 (38.78)	391 (73.91)	0.22 (0.12, 0.41)	<0.001
Heard of PrEP					
No	455 (78.72)	36 (73.47)	419 (79.21)	1.00	
Yes	123 (21.28)	13 (26.53)	110 (20.79)	1.38 (0.71, 2.68)	0.350
Uptake of PrEP					
No	558 (96.54)	39 (79.59)	519 (98.11)	1.00	
Yes	20 (3.46)	10 (20.41)	10 (1.89)	13.31 (5.23, 33.90)	<0.001
Acceptance of PrEP					
No	130 (22.49)	14 (28.57)	116 (21.93)	1.00	
Yes	448 (77.51)	35 (71.43)	413 (78.07)	0.70 (0.37, 1.35)	0.289

**Table 5:** Factors associated with acceptance of MC and PrEP among MUSs.

Variables	*B*	S.E.	Wald	AOR (95% CI)	*p* value
*MC*					
Registered residence					
Nanning city				1.00	0.052
Cities besides Nanning in Guangxi province	0.292	0.250	1.373	1.34 (0.82, 2.19)	0.241
Provinces besides Guangxi in China	0.928	0.382	5.894	2.53 (1.20, 5.35)	0.015
Medical students	0.631	0.256	6.084	1.88 (1.14, 3.10)	0.014
MSM	-0.969	0.382	6.430	0.38 (0.18, 0.80)	0.011
MC surgery					
No				1.00	<0.001
Yes	1.357	0.501	7.329	3.89 (1.46, 10.38)	0.007
Unknown	-2.264	0.344	43.331	0.10 (0.05, 0.20)	<0.001
*PrEP*					
Time of residence in Nanning					
1 year				1.00	0.010
1~2years	-0.502	0.260	3.737	0.61 (0.36, 1.01)	0.053
>2 years	-0.767	0.260	8.679	0.47 (0.28, 0.77)	0.003
Comprehension of MSM					
Unchangeable	0.290	0.268	1.170	1.34 (0.79, 2.26)	0.279
Treatable	-0.335	0.327	1.051	0.72 (0.38, 1.36)	0.305
Infectious	-0.643	0.312	4.249	0.53 (0.29, 0.97)	0.039
Normal				1.00	0.042
Nationality					
Han				1.00	0.043
Zhuang	0.750	0.306	6.023	2.12 (1.16, 3.86)	0.014
Other minorities	-0.131	0.461	0.081	0.88 (0.36, 2.16)	0.775
Frequency of alcohol consumption					
Never				1.00	0.038
Occasional	-0.374	0.224	2.781	0.69 (0.44, 1.07)	0.095
<250 ml/day	-1.568	0.604	6.730	0.21 (0.06, 0.68)	0.009
250-500 ml/day	-0.507	0.628	0.651	0.60 (0.18, 2.06)	0.420
>500 ml/day	-0.980	0.526	3.472	0.38 (0.13, 1.05)	0.062
Uptake of PrEP before	-1.503	0.533	7.960	0.22 (0.08, 0.63)	0.005

## Data Availability

The datasets generated and/or analyzed during the current study are not publicly available due to ethical and legal reasons but are available from the corresponding author on reasonable request.

## Supplementary Materials

Supplementary Materials Questionnaire on male university students' knowledge, attitude, and practice of male homosexuality in Nanning universities.
