# Supplementary material for: A Survey on HIV/AIDS-Related Knowledge, Attitudes, Risk Behaviors, and Characteristics of Men Who Have Sex with Men among University Students in Guangxi, China
Source: Biomed Res Int. 2020 Jun 14;2020:7857231. doi: 10.1155/2020/7857231 (PMC7312710; doi:10.1155/2020/7857231)
Supplement: Supplementary Materials — Questionnaire on male university students' knowledge, attitude, and practice of male homosexuality in Nanning universities. [file 7857231.f1.docx]

**Questionnaire on male university students’ knowledge, attitude and practice of male homosexuality in Nanning universities**

Dear schoolmates：

Hope you have a good day! We are students from School of Public Health, Guangxi Medical University. The purpose of this questionnaire is to survey knowledge, attitude and behavior of Nanning male university students related to men who have sex with men (MSM). This is an anonymous questionnaire only for research use, we will strictly confidential for personal circumstances of respondents, please be assured complete. Thank you for your assistance and support!

Note: 1. Please tick "√" in "□" before the appropriate option.

2. All options are single (except those with special instruction).

| **A Basic Information** | |
| --- | --- |
| **University:** | **Grade:** |
| Major: | Date of birth (YYYY/MM/DD): |
| Nationality : □Han □Other (Please specify:_______________) | |
| Registered residence: Guangxi：□Nanning City □Other city (Please specify:____________)  □Other Province (Please specify:____________) | |
| Time of residence in Nanning： □＜3 months □3-6 months  □7-12 months □1 – 2 years □ >2 years | |
| Which of the following is your sexual preference?  □Homosexuality □Heterosexuality □Double sexuality □Uncertain | |

| **B HIV and MSM related Knowledges** | | |
| --- | --- | --- |
| B01 | Can a person infected with HIV be seen from his appearance? | □Yes □No □I don’t know |
| B02 | Can mosquito bites spread HIV? | □Yes □No □I don’t know |
| B03 | Can you be infected with HIV by eating with someone who is HIV positive? | □Yes □No □I don’t know |
| B04 | Can you be infected with HIV by transfusing blood with HIV? | □Yes □No □I don’t know |
| B05 | Can you be infected with HIV by sharing needles and injector with someone who is HIV positive? | □Yes □No □I don’t know |
| B06 | Is it possible for a child be infected with HIV if the mother was HIV positive when delivery? | □Yes □No □I don’t know |
| B07 | Can the correct use of condoms reduce the spread of HIV? | □Yes □No □I don’t know |
| B08 | Can having sex with just one partner reduce the spread of HIV？ | □Yes □No □I don’t know |
| B09 | What are the main sources of your HIV/AIDS related information? (multiple choice) | □Television broadcasting □The press □Books □Friends □Doctors □Consultation service (including telephone consultation) □Free promotional materials □Publicity board and advertising board □Internet □School education □Other (Please specify:_______________) |
| B10 | Why are MSM more likely to get HIV? (multiple choice) | □Semen contains a lot of virus, and anal sex is prone to rupture bleeding, the HIV in semen can rupture into the body through the wound  □Gay men usually have multiple sex partners, which may increase the chance of HIV transmission |
| B11 | Which do you think is the most appropriate description of male homosexuality? | □A normal emotional choice  □An alternative fashion trend  □An abnormal psychological distortion  □An act that endangers the society |
| B12 | You think MSM are： | □unchangeable  □treatable  □infectious  □normal |

| **C HIV and MSM related Attitudes** | | |
| --- | --- | --- |
| C01 | Are you afraid of AIDS? | □Very □A little □No |
| C02 | What do you think your risk of infected by HIV? | □Never □Low □High |
| C03 | Have you ever had an HIV test? And what was the result? | □Yes, it was positive (Jump to C05)  □Yes, it was negative (Jump to C05)  □Yes, but I don’t know the result (Jump to C05)  □No |
| C04 | Why didn’t you test for HIV? | □Not necessary  □Fear of others knowing my test result  □Fear of knowing the test result  □I don't know where to test |
| C05 | What will you do if a family member or friend of yours is infected with HIV? (multiple choice) | □Get away from them  □Help them  □None of my business  □Be willing to get along with them □They should be discriminated  □Keep the secret for them  □Their condition should be publicized to prevent HIV transmission |
| C06 | What do you think of the relations between men homosexuality? | □No different with heterosexuality  □Weird  □Dirty |
| C07 | Is sexual interaction between MSM a normal sexual need? | □Strongly disagree □Agree  □Neutrality □Disagree |
| C08 | Can you accept living or working with an HIV-infected person? | □Yes □No  □I don’t know |
| C09 | How much do you know about MSM? | □A lot □So so  □A little □Not at all（Jump to C11） |
| C10 | How did you learn about MSM? | □Newspapers and magazines  □Film and television works  □Lessons in school  □Communication with classmates and friends  □Other (Please specify:_______________) |
| C11 | What do you think is the main reason why the society doesn't accept male homosexuality? | □Influence of traditional senses  □It increasing the spread of HIV  □I don’t know |
| C12 | What would your attitude be if the society accepted male homosexuality one day? | □Respect □Hate □Neutrality |
| C13 | Does your school offer health education on homosexuality? | □Yes □No |
| C14 | Do you think it is necessary for schools to strengthen health education on homosexuality? | □Yes (Jump to C16) □No |
| C15 | Why do you think it’s not necessary for schools to strengthen health education on homosexuality? | □It’s none of my business  □I’ve gotten sufficient related knowledge  □Health education will not solve the serious problem of HIV infection among gay men |
| C16 | Can you accept MSM? | □Yes □No (Jump to C18) |
| C17 | Why can you accept MSM? (multiple choice) | □I shouldn't interfere in other people's lives (Jump to C19)  □Undying love deserves respect in all its forms (Jump to C19)  □Gay men are harmless to other people (Jump toC19) |
| C18 | Why can’t you accept MSM? (multiple choice) | □MSM endanger people’s lives and the society  □MSM spread HIV  □MSM are disgusting  □MSM are in an abnormal state of mind |
| C19 | What would your attitude be if you found some of your acquaintances are MSM? | □Interested  □Indifferent  □Uncomfortable  □Aloof |
| C20 | What would you do if you fell in love with a male? | □Keep on  □Restrain  □Feel panic |
| C21 | Should the rights of MSM be given care and protection? | □Should give special attention and protection  □Should give some attention and protection  □Should not give any attention and protection  □It depends |

| **D HIV and MSM related Behaviors** | | |
| --- | --- | --- |
| D01 | Have you ever smoked in the recent six months? | □Yes □No (Jump to D03) |
| D02 | How much did you smoke in the recent six months? | □Occasionally  □<10 cigarettes/day  □10–20 cigarettes/day  □>20 cigarettes/day |
| D03 | How much did you drink in the recent six months? | □Occasionally □<250 ml/day  □250–500 ml/day □>500 ml/day  □Never |
| D04 | Have you ever injected drugs in the recent six months? | □Yes □No (Jump to D06) |
| D05 | What did you inject? | □Heroin  □Methamphetamine  □Cocaine  □Other (Please specify:_______________) |
| D06 | Have you ever thought about having sex with a man? | □Yes □No (Jump to D08) |
| D07 | Why did you think so? (multiple choice) | □Inspired by film and television works  □Inspired by related forums on Internet  □Inspired by other people  □Other (Please specify:_______________) |
| D08 | Was your first sexual partner a male or a female? | □Male □Female (Jump to D11)  □No sexual experience (Jump to D22) |
| D09 | How old were you when you had sex with a man at the first time? | □___________years old □I can't remember  Were you：□Unforced □Forced |
| D10 | What was your first male sexual partners? | □An old man  □A middle age man  □Contemporary man  □Other (Please specify:_______________) |
| D11 | Are your sexual partners fixed? | □Yes □No |
| D12 | Have you had sex with a man in the recent six months? | □Yes □No (Jump to D19) |
| D13 | How many different men have you had sex with in the recent six months? | □________men □I don’t remember |
| D14 | What kind of sexual intercourse have you had with men in the recent six months? | □Anal intercourse □Oral intercourse  □Both □Other |
| D15 | Are you a receptive partner or an insertive one when you have anal intercourse with a man? | □Receptive partner □Insertive partner  □Both |
| D16 | Where have you had sex in the recent six months? | □Toilet □Dormitory □Hotel □Other (Please specify:_______________) |
| D17 | How often have you used condoms during anal intercourse with men in the recent six months? | □Never □Sometimes  □Every time |
| D18 | Have you ever had sex with man by pay in the recent six months? | □Yes □No |
| D19 | Have you ever had sex with woman in the recent six months? | □Yes □No (Jump to D22) |
| D20 | How many different women have you had sex with in the recent six months? | □__________women □I don’t remember |
| D21 | How often have you used condoms when had sex with women in the recent six months? | □Never □Sometimes  □Every time |
| D22 | Have you ever done circumcision? | □Yes □No □I don’t know |
| D23 | Are you willing to circumcise if circumcision can prevent AIDS? | □Yes □No |
| D24 | Have you ever used PrEP? | □I’ve never heard □Yes, I have  □No, I haven’t |
| D25 | Are you willing to take PrEP if it can prevent AIDS? | □Yes □No |

**This is the end of the questionnaire. Thank you for your cooperation！**
